# Buffalo Medical College—Session of 1882–83

**Published:** 1882-10

**Authors:** 


### Buffalo Medical College.

### *Session of 1882–83.*

The introductory lecture to the present session was given by Prof. R. A. Witthaus, on Wednesday evening, Sept. 27th, at the college building. Dr. Witthaus has been elected to the Chair of Chemistry, made vacant by the resignation of Dr. Doremus. The subject of his first lecture was, "The Relations which Chemical Force bears to the other Forces of Nature and to the production of Animal Force." The lecture was illustrated by numerous experiments, and made a favorable impression upon the class and the audience.

We learn that Dr. Miner, long connected with this journal and with the college, has been compelled, by the state of his health, to resign the Chair of Special and Clinical Surgery. Prof. Moore will add the work and duties of the position thus vacated to his labors during the present session.

